# National survey of outcomes and practices in acute respiratory distress syndrome in Singapore

**DOI:** 10.1371/journal.pone.0179343

**Published:** 2017-06-16

**Authors:** Shahla Siddiqui, Zudin Puthucheary, Jason Phua, Benjamin Ho, Jonathan Tan, Siau Chuin, Noelle Louise Lim, Chai Rick Soh, Chian Min Loo, Addy Y. H. Tan, Amartya Mukhopadhyay, Faheem Ahmed Khan, Azman Johan, Aik Hau Tan, Graeme MacLaren, Juvel Taculod, Blesilda Ramos, Tun Aung Han, Matthew E. Cove

**Affiliations:** 1 Khoo Teck Puat Hospital, Yishun, Singapore, Singapore; 2 Departments of Medicine, Anaesthesia and Surgery, National University Hospital, National University Health System, Singapore, Singapore; 3 Centre for Human Health and Performance, University College London, London, United Kingdom; 4 Departments of Medicine and Anaesthesia, Tan Tock Seng Hospital, Singapore, Singapore; 5 Department of Medicine and Anaesthesia, Changi General Hospital, Singapore, Singapore; 6 Department of Medicine and Anaesthesia, Singapore General Hospital, Singapore, Singapore; 7 Department of Critical Care, Ng Teng Fong General Hospital, Jurong Health, Singapore, Singapore; 8 School of Nursing, Ngee Ann Polytechnic, Singapore, Singapore; Azienda Ospedaliero Universitaria Careggi, ITALY

## Abstract

**Introduction:**

In the past 20 years, our understanding of acute respiratory distress syndrome (ARDS) management has improved, but the worldwide incidence and current outcomes are unclear. The reported incidence is highly variable, and no studies specifically characterise ARDS epidemiology in Asia. This observation study aims to determine the incidence, mortality and management practices of ARDS in a high income South East Asian country.

**Methods:**

We conducted a prospective, population based observational study in 6 public hospitals. During a one month period, we identified all ARDS patients admitted to public hospital intensive care units (ICU) in Singapore, according to the Berlin definition. Demographic information, clinical management data and ICU outcome data was collected.

**Results:**

A total of 904 adult patients were admitted to ICU during the study period and 15 patients met ARDS criteria. The unadjusted incidence of ARDS was 4.5 cases per 100,000 population, accounting for 1.25% of all ICU patients. Most patients were male (75%), Chinese (62%), had pneumonia (73%), and were admitted to a Medical ICU (56%). Management strategies varied across all ICUs. In-hospital mortality was 40% and median length of ICU stay was 7 days.

**Conclusion:**

The incidence of ARDS in a developed S.E Asia country is comparable to reported rates in European studies.

## Introduction

The term “Acute Respiratory Distress Syndrome” (ARDS), was first used in 1967 to describe a series of mechanically ventilated patients who developed refractory hypoxia, diffuse pulmonary infiltrates and reduced compliance [1]. Since then, our understanding of the underlying pathophysiology has greatly improved, particularly the role of the ventilator [2]. However, the world-wide incidence remains poorly characterized. The wide range quoted, 5–64 per 100,000 persons per year [3,4], may reflect regional variation in health care practices and different interpretations of ARDS definitions [4,5]. Despite this regional variation, there are very little data characterising the epidemiology of ARDS in Asian countries. Furthermore, the impact of introducing a new ARDS definition in 2012 has not been evaluated in large nationwide studies. As a result, the incidence of ARDS following the newer definition is unknown [6].

Determining the incidence and outcomes for ARDS is necessary for appropriate intensive care resource planning. ARDS management is complex and consumes considerable health resources. In 1999, adult ARDS patients in the USA cost $57,000 per admission and accounted for 2.7 million hospital days [4,7]. In addition, more that 50% of ARDS patients are unable to return to work at 12 months, which may burden societal resources [8]. The current cost of managing ARDS is unknown, but may vary between centres, particularly with the recent increase in the reported use of extracorporeal membrane oxygenation (ECMO) for respiratory failure [9].

In this prospective multicentre study we aim to describe the incidence, outcomes, cost and management of ARDS by conducting a nationwide study in a small, high income, Asian country. We hypothesised that these characteristics would be no different to those observed in other high income countries.

## Materials and methods

### Study design and setting

We conducted a national, prospective observational study over a period of 4 weeks, starting on May 15^th^ 2015, to determine the incidence and outcomes of ARDS in Singapore. Healthcare in Singapore is provided by 6 regional health sectors, each with one or more public hospitals. In this study, we included the major adult public hospital in each of these 6 sectors. The hospitals ranged in size from 590 to 1500 beds and represented a total of 132 ICU beds. Two public hospitals were not included in the study, a women and children’s hospital and a psychiatric hospital. Principle investigators at each of the 6 hospitals were identified and contacted through the Society of Intensive Care Medicine in Singapore.

### Patient identification and data collection

After obtaining Institutional Review Board approval to collect data with waiver of consent from Singapore’s National Healthcare Group Domain Specific Review Board (DSRB 2015/00365), all mechanically ventilated patients admitted during the study period were screened daily for development of ARDS using the Berlin definition criteria [10]. Patients were included in the study if they were admitted to ICU during the study period, received ventilator support, were at least 21 years old, and fit the new Berlin criteria for ARDS: i.e. an arterial partial pressure of oxygen (PaO_2_) to fraction of inspired oxygen (FiO_2_) ratio of 300 or less, bilateral infiltrates on chest x-ray (CXR) and a positive end expiratory pressure (PEEP) of 5 cmH_2_O or more. Onset of ARDS was defined as the first day the patient met all ARDS criteria. Clinical and physiological data was collected for the first 24 hours of admission to determine APACHE II score. Ventilation parameters were collected at the time ARDS criteria were met, as well as ventilation parameters corresponding to the highest FiO_2_ ratio. Hospital mortality and cost data were also collected.

### Outcome measures and statistical analysis

The primary aim was to determine the incidence of adult ARDS expressed as cases per 100,000 inhabitants per year in the population aged 21 years and older. The catchment population was determined using published resident population figures from the government of Singapore (3,902,690) [11]. Age adjusted incidence rate was calculated using age specific resident population data. The median age in Singapore is 39.6 years, 22% are below the age of 21 (0.9 million) and 11.8% are aged 65 years, or older, just under half the residents are male (49%). Ethnically, the population is mostly composed of persons of Chinese descent (74.3%), with those of Malay and Indian descent accounting for 13.3% and 9.1% respectively [11]. Data are reported as percentage, mean ± standard deviation and median with interquartile range (IQR) where appropriate. Differences between continuous variables were analysed using Students T test or Wilcoxon rank-sum test, for normal or parametric distributions respectively. Distributions of categorical data were compared using Pearson’s χ^2^ test. All data analysis was performed using STATA^TM^ version 13 *(StataCorp*, *Texas*, *USA)*, a p-value < 0.05 was considered significant.

## Results

From May 15^th^ to June 15^th^ 2015, 1200 ICU admissions occurred, of which 904 patients (73%) required mechanical ventilation for at least 24 hours. Fifteen patients had ARDS (1.25% of ICU admissions), 7 with moderate ARDS and 8 with severe ARDS. No patients met criteria for mild ARDS, but oxygenation data is missing for one patient. The median age was 59 (IQR 52–70), and nearly three quarters (73%) of the patients were male (Table 1). Most of the patients were ethic Chinese (63%), but a quarter were Malay and all Malay patients developed severe ARDS (Table 1). Median body mass index on admission was 22.8 (IQR 20.3–26.4) and the median Apache II score was 33 (IQR 25–40). Most patients were diagnosed as having ARDS due to pneumonia (79%). All patients with ARDS secondary to non-pulmonary sepsis developed severe ARDS.

**Table 1:** Demographics and general clinical data. Unless otherwise indicated median is presented and value ranges in brackets represent interquartile range (IQR).

Variable	Total (15)	Moderate ARDS (7)	Severe ARDS (8)	p-value
Age	59 (52–70)	62 (48–71)	59 (51–65)	0.52
Male Gender, n (%)	11 (73)	5 (70)	6 (75)	0.88
Race				
Chinese, n (%)	9(63)	6 (86)	3 (37.5)	0.073
Malay, n (%)	4 (26)	n/o	4 (50)	
Indian, n (%)	1 (7)	n/o	1 (12.5)	
Other, n (%)	1(7)	1 (14)	n/o	
Body Mass Index (IQR)	22.8 (20.3–26.4)	25 (19–27)	23 (20–22)	0.75
APACHE II score (IQR)	33 (25–40)	33 (22–38)	33 (28–40)	0.64
PaO_2_:FiO_2_ ratio at onset (IQR)	93 (80–115)	115 (102–115)	83 (48–90)	
PEEP (cmH_2_O) at onset (IQR)	10 (8–10)	10 (8–12)	10 (5–10)	0.63
Causes of ARDS^§^				
Pneumonia, n (%)	11 (69)	6 (86)	5 (71)	0.21
Trauma, n (%)	1 (7)	1 (14)	n/o	
Sepsis, non-pulmonary, n (%)	2 (14)	n/o	2 (29)	
Ventilation first 24 hours				
V_T_, ml/kg PBW*^‡^ (IQR)	7.5 (6.2–9)	6.5 (6.2–8.3)	8.15 (6.9–9.5)	0.27
>8ml/kg PBW*^‡^, n (%)	6 (38)	2 (28)	4 (50)	0.40
FiO_2_ (IQR)	0.8 (0.6–1.0)	0.65 (0.6–0.8)	0.9 (0.8–1.0)	0.23
Peak insp. pressure cmH_2_O^‡^ (IQR)	32 (30–36)	31 (30–32)	36 (32–36)	0.14
Plateau pressure cmH_2_O^‡^ (IQR)	29 (27–32)	33.5 (29–38)†	29 (27–30)	0.33
PEEP cmH_2_O^‡^ (IQR)	15 (10–16)	14 (12–20)	15 (10–15)	0.35
Driving pressure cmH_2_O^¶^ (IQR)	20 (7–22)	9.5 (1–18)†	22 (20–22)	0.12
Rescue strategies				
Prone positioning, n (%)	2 (13)	n/o	2 (25)	0.16
NMB, n (%)	1 (7)	n/o	1 (13)	0.33
Corticosteroids, n (%)	1 (7)	1 (14)	n/o	0.27
ECMO Referral, n (%)	1 (7)	n/o	1 (13)	0.33

n/o = not observed.

§ Data missing for one patient.

* PBW = Predicted body weight.

‡Value at ARDS onset.

¶ Driving pressure is defined as difference between Plateau pressure and PEEP. Peak insp. pressure = peak inspiratory pressure.

† Plateau pressure only available for 2 patients.

NMB = neuromuscular blockers.

At the onset of ARDS, median P/F ratio was 93 (IQR 80–115) and median PEEP was 10 cmH_2_O (IQR 8–10), increasing to 15 cmH_2_O (IQR 10–16) in the first 24 hours. Median tidal volume was 7.5 ml/kg predicated body weight (PBW), but more than one third of the patients (38%) received a tidal volume exceeding 8 ml/kg PBW. Median plateau pressure (Pplat) was 29 cmH_2_O with a corresponding median driving pressure (Pplat—PEEP) of 20 cmH_2_O. Only 2 patients were nursed in the prone position, 1 patient received neuromuscular blockers and 1 patient was referred for Extracorporeal Membrane Oxygenation (Table 1). Total in-hospital mortality was 40% (Table 2). The overall, unadjusted, incidence of ARDS was 4.5 per 100,000 population per year and the age-adjusted incidence 5.8/100,000 population per year (Table 2). The median cost, inclusive of subvention, was US$51,730 (IQR US$31,530 –US$90,890). A STROBE checklist and a copy of the anonymised baseline data is attached as S1 and S2 Files. Original data may be requested from the domain specific review board.

**Table 2:** Summary of the main studies reporting ARDS epidemiology published since 2000.

	Current study	LUNG-SAFE [6]	ALIEN Study [5]	Li et al [17]	Linko et al [13]	ICCTG^¶^ [31]	Rubenfeld et al [4]	Bersten et al [14]
Country	Singapore	Multiple^#^	Spain	USA	Finland	Ireland	USA	Australia
Study design	Prospective	Prospective	Prospective	Retrospective	Prospective	Prospective	Prospective	Prospective
Study duration	1 month	4 weeks	1 year	8 years	8 weeks	10 weeks	12 months	2 months
Study Period	May-Jun 2015	Winter 2014	Nov 2008-Oct 2009	Jan 2001—Dec 2008	Apr-Jun 2007	Aug—Oct 2006	Apr 1999—Jul 2000	Oct—Nov 1999
Number of hospitals	6	435	17	2	18	N/A	21	N/A
Number of ICUs	13	459	354	N/A	25	14	N/A	21
Catchment population	3,902,690	N/A	3,546,629	124,277	4,164,980	N/A	1,740,000	2,941,137
Number of ICU beds	132	N/A	354	164	N/A	N/A	430	253
ICU beds/100,000 population	5.0	N/A	8.2	132	N/A	N/A	24.7	8.6
ICU admissions	1,200	29,144	11,363	8,034	2,670	1,029	N/A	1,977
Number receiving MV	904	12,906	3,462	N/A	958	728	6235	N/A
Total ARDS cases	15	2,813	255	514	32	196	828	148
PaO_2_:FiO_2_	93 (80–115)^†^	161 (158–163)^‡^	114 (104–124)^‡^	N/A	200 (138–275)	170 (160–180)^‡^^§^	N/A	176 (166–186)^‡^
ICU incidence of ARDS per ICU bed*	0.10	0.42	0.06	0.03	N/A	N/A	0.14	0.27
Unadjusted ARDS cases/100,000 population	4.5	N/A	5.9	N/A	N/A	N/A	58.7	N/A
Adjusted ARDS cases/100,000 population	5.8	N/A	7.2	38.3 (in 2008)	5.0	N/A	64.0	28
Mortality	40%	35.3%	47.8%	16% (in 2008)	47%	32.3%	41.1%	34%

¶ Irish Critical Care Trails Group.

# Data from 48 countries.

* Number of ARDS cases per ICU bed, normalized to 4 weeks.

† Median (IQR).

‡ Mean (95% Confidence interval).

§ Converted from kPa. N/A = Not available in reported data.

## Discussion

This is the first nationwide study of ARDS in Southeast Asia. The age-adjusted incidence is comparable to European studies (Table 2), where the reported incidence ranges from 4.9–13.5/100,000 person-years [3,5,6,12,13]. However, similar to European studies, this incidence differs from studies in Australia, New Zealand and the USA [4,14]. The reasons for this disparity remain unclear, but are likely influenced by a variety of factors.

First, the incidence may be affected by interpretation of the definitions, as well as evolution of the definitions over time [4]. Second, interpretation of chest roentgenogram can vary significantly, with intensivists agreeing less than half the time on the presence of bilateral infiltrates [15,16]. Third, seasonal variations in climate may affect the incidence of respiratory illnesses associated with ARDS, though regional variation was preserved in studies covering a year or more [4,5,17]. Fourth, the proportion of ICU beds/100,000 population in the United States is three times the number of beds in Europe and Singapore (Table 2). Countries with high ICU bed availability admit a larger proportion of hospitalised patients to ICU, who tend to be older and have more chronic medical conditions [18]. These characteristics are reported risk factors for ARDS [19].

Our ARDS incidence per ICU bed was much lower than the Asian components of the recent Lung-safe study, where an incidence of 0.27 cases/ICU bed over 4 weeks was seen [6]. However, data collection for Lung-safe occurred during winter, possibly inflating the incidence. Although Singapore has no distinct seasons, an increase in incidence of respiratory infections correlates with winter in either hemisphere [20] and we cannot exclude a higher incidence if data collection had been timed differently.

A surprising observation was the fact that all Malay patients developed severe ARDS, although the overall distribution of race between moderate and severe ARDS patients was not significantly different (Table 1). Whether this represents random variation, genetic susceptibility, socioeconomic differences, or a combination is unclear and warrants further investigation. Race plays an important, complex, role in the presentation of illness [21]. Another surprising observation was the low utilisation rate of rescue interventions. However, we did not collect information regarding contraindications to prone positioning or neuromuscular blockade. In this study we were particularly interested to determine if the cost of managing ARDS patients has increased, since ECMO is now used more frequently. However, only one patient received ECMO during the study period, and our median cost was US$51,730, which is similar to reported costs before adult ECMO use increased [22].

In our study we found that median ventilation settings were consistent with lung protective ventilation (Table 1), but we also identified levels of non-compliance, where over one third of patients received tidal volumes which are not considered protective. Similar findings have been reported in large registry studies in multiple countries [23–28]. Furthermore, driving pressure, a surrogate measure of over distention, was higher than that considered physiological and safe [29]. It is clear from both our study, and others [23–28], that achieving ventilation parameters consistent with lung protective settings in all ARDS patients remains a challenge for intensivists. It is therefore not surprising that the mortality in our study is similar to that reported elsewhere (Table 2).

Our study has several limitations. First, we did not detect any patients mild ARDS during the study period. There is evidence that mild ARDS is under-recognised. In the recent Lung-safe study recognition rates of only 51.3% were seen for mild ARDS, compared with rates of 78.5% for severe ARDS [6]. It is possible that the reliance of the wider multi-disciplinary team to identify ARDS may have introduced susceptibility to clinician under-recognition of mild ARDS. Second, 10 private intensive care units did not participate. These hospitals account for 24% of total hospital admissions, but the population served by the private sector are predominantly the non-resident, expatriate community, who are not included in resident population counts. Third, we collected data for only one month. Under ideal circumstances, we would have tracked the incidence over 12 months or more, but the resources were not available. Several other ARDS studies have been conducted over periods of 10 weeks or less [6,13,30,31], and this may partly explain the variability in published incidence rates.

## Conclusion

The adjusted ARDS incidence rate of 5.8/100,000 population in Singapore is similar to incidence rates reported in Europe. Compliance with lung protective ventilation was suboptimal and may reflect the international difficulties in translating research into practise.

## Supporting information

S1 File STROBE checklist. (PDF)

S2 File Baseline anonymised data. (XLS)
